# Microbial Remobilisation on Riverbed Sediment Disturbance in Experimental Flumes and a Human-Impacted River: Implication for Water Resource Management and Public Health in Developing Sub-Saharan African Countries

**DOI:** 10.3390/ijerph14030306

**Published:** 2017-03-15

**Authors:** Akebe Luther King Abia, Chris James, Eunice Ubomba-Jaswa, Maggy Ndombo Benteke Momba

**Affiliations:** 1 Departments of Biotechnology, Vaal university of Technology, Private Bag X021, Andries Potgieter Blvd, Vanderbijlpark 1911, South Africa; lutherkinga@yahoo.fr; 2 School of Civil & Environmental Engineering, University of the Witwatersrand, Johannesburg 2050, South Africa; Chris.James@wits.ac.za; 3 Natural Resources and the Environment, CSIR, P.O. Box 395, Pretoria 0001, South Africa; eubombajaswa@csir.co.za; 4 Department of Environmental, Water and Earth Science, Tshwane University of Technology, Arcadia Campus, 175 Nelson Mandela Drive, Pretoria 0001, South Africa

**Keywords:** *Escherichia coli*, sediment microbial quality, riverbed sediment disturbance, sediment resuspension, public health risk, water resource management, alternative water sources, water resources monitoring

## Abstract

Resuspension of sediment-borne microorganisms (including pathogens) into the water column could increase the health risk for those using river water for different purposes. In the present work, we (1) investigated the effect of sediment disturbance on microbial resuspension from riverbed sediments in laboratory flow-chambers and in the Apies River, Gauteng, South Africa; and (2) estimated flow conditions for sediment-borne microorganism entrainment/resuspension in the river. For mechanical disturbance, the top 2 cm of the sediment in flow-chambers was manually stirred. Simulating sudden discharge into the river, water (3 L) was poured within 30 s into the chambers at a 45° angle to the chamber width. In the field, sediment was disturbed by raking the riverbed and by cows crossing in the river. Water samples before and after sediment disturbance were analysed for *Escherichia coli*. Sediment disturbance caused an increase in water *E. coli* counts by up to 7.9–35.8 times original values. Using Shields criterion, river-flow of 0.15–0.69 m^3^/s could cause bed particle entrainment; while ~1.57–7.23 m^3^/s would cause resuspension. Thus, sediment disturbance in the Apies River would resuspend *E. coli* (and pathogens), with possible negative health implications for communities using such water. Therefore, monitoring surface water bodies should include microbial sediment quality.

## 1. Introduction

In recent years, the accumulation of microorganisms including pathogens in riverbed sediments and possible resuspension during human-induced activities or natural processes has received increased research attention worldwide [1,2,3,4,5]. Once in the aquatic environment, a number of processes could lead to the settling of microorganisms and their subsequent resuspension from the bed sediments. Such processes could include natural gravitation, hyporheic exchange with the waterbed, attachment to suspended particles and aquatic vegetation, and filtration within the bed sediments [6,7,8,9,10]. Attachment to sediment particles could be through weak reversible van der Waals forces or strong irreversible forces due to the secretion of extracellular polymeric substances (EPS) and other bacterial appendages [7,11]. The bacteria could also be found in inter-sediment spaces within the riverbed sediments [12,13]. Increased flow within a water catchment may lead to erosion of riverbed sediments [14,15,16]. As reviewed by Chang and Scotti [17], sediment resuspension from the riverbed is determined by the intensity of the river-flow above the bed. When the river-flow exerts stress on the riverbed above a certain critical value, bed materials in contact with the water begin a rolling or jumping motion along the bed surface (entrainment). When the stress exerted by the flowing river increases further above the entrainment conditions, this may lead to turbulence at the sediment–water boundary layer, resulting in bed particles being lifted away from the riverbed (resuspension) and transported as suspended load [17,18,19]. This movement results in the re-suspension of bacteria from the sediment layer [20,21,22,23,24]. Once resuspended, the microorganisms could exist in a free-living state [25,26] or could resettle through the processes mentioned earlier.

Extreme storm events cause sediment resuspension due to increase in flow, while prolonged drought events may lead to discontinuity of water supply from the main distribution systems in many water-scarce countries. As a result, there is an increased use of alternative water bodies such as rivers (e.g., for cleaning, bathing, and drinking). The increased use of rivers may result in the disturbance of riverbed sediments and consequently increase the human health risk associated with exposure to resuspended enteric waterborne pathogens [27,28,29]. This resuspension could also increase the cost involved in treating such water for human use.

South Africa is not excluded from the current tremendous world population growth. With a current population of over 54 million, the country has experienced an increase in annual population growth rate from 1.27% in 2002 to 1.58% in 2014 [30] with tremendous pressure being placed on resources and infrastructure. Rural communities have been the most affected by this increase as most of them are located in areas with limited or complete lack of potable water supply, adequate sanitation, and waste management facilities [31]. As in many developing countries, these communities resort to surface water (especially rivers) as main or alternative water sources despite their low microbial quality [32,33,34,35]. South Africa’s surface waters are also used for recreational activities, especially during the hot summer months. Recent studies have demonstrated the presence of higher concentrations of *Escherichia coli* in riverbed sediments compared to the water column [36]. Genes for pathogenic organisms like *Salmonella* spp., *Shigella* spp. and *Vibrio cholerae* have also been detected in riverbed sediments in South Africa, indicating the possible presence of these pathogenic organisms [37]. In a study conducted by Abia et al. [38], the authors demonstrated through laboratory experiments that these pathogens could survive in the sediments of the Apies River for up to 30 days. Despite this finding, current monitoring of the country’s water resources (as with many developing countries) for microbial quality does not take into consideration sediment quality [39].

Although previous studies have demonstrated riverbed resuspension, this has mostly been in relation to recreational activities in developed countries. Sediment resuspension in rivers that are directly and extensively used for personal and household hygiene as well as drinking, has not been given much attention. In addition, most studies have employed complex approaches to demonstrate the process of resuspension of sediment-borne microorganisms [1,20,23,24]. As such, this paper reports on simple, but effective, experiments on hydraulic and mechanical disturbances of sediment in both flumes and at reach scale in a river. The aim of the study was to (1) investigate the possible impact of mechanical disturbance and increased river flow on the resuspension of *E. coli* from riverbed sediments of the Apies River, Gauteng, using laboratory and field experiments; and (2) to estimate the flow conditions necessary for entrainment and resuspension of particles from the riverbed. To our knowledge, this study is the first to assess the impact of sediment disturbance on the microbial quality of river water in South Africa. The results of this study will be a useful contribution and could be quite influential in promoting increased awareness of the microbial health hazard associated with riverbed sediments—a hazard that can come to occur with mobilisation by flood waves or disturbance by people or livestock. Such information could be of great significance to water governing bodies within South Africa and other Sub-Saharan countries, as disturbance of microbially-contaminated river sediments could have negative impacts on public health.

## 2. Materials and Methods

### 2.1. Study Site

**Figure 1:**
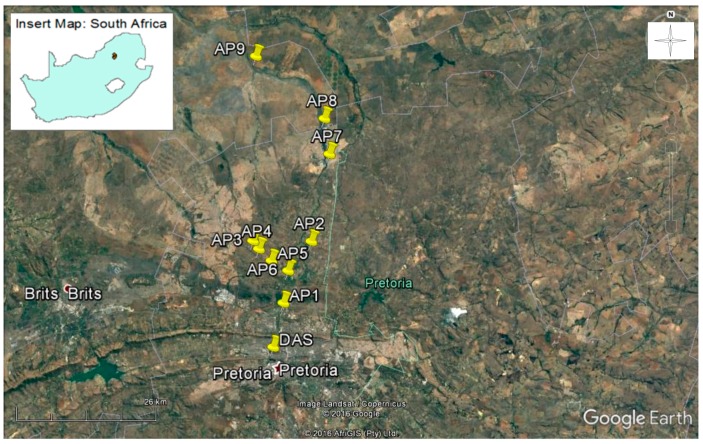
Map of study sites (Source: Google Earth). DAS: wastewater treatment plant; AP1–AP4, AP8: sites included in entrainment/resuspension parameters estimation; AP5, AP6, AP7: sediment and water collection sites for laboratory experiments; AP6 (raking site) and AP9 (cow-crossing site): field experiments.

This study was carried out in the Apies River that flows across the city of Pretoria in the Province of Gauteng, South Africa (Figure 1).

The study site (Apies river) has previously been described [36,40]. Located in a catchment characterised by high erodibility, the river receives high sediment loads, especially during run-off in the wet season. Extensive industrialisation and increasing human settlements along the river has led to the destruction of the vegetation around the river and canalisation of most parts of the river within urban areas. The presence of weirs and a dam on the river’s course affects the flow of the river in some areas. However, other parts of the river, especially as it passes through the rural and agricultural areas, have been maintained in their natural state. These morphological alterations along the river, therefore, lead to erosion in narrow areas of high flow and deposition in broader areas with low flow. The river water is used by surrounding communities for irrigation, watering of cattle, household and personal hygiene, spiritual cleansing and fishing. In some informal settlements, the river is the only water source available for the inhabitants and at the same time serves as a point for human waste disposal.

### 2.2. Laboratory Experiments

**Figure 2:**
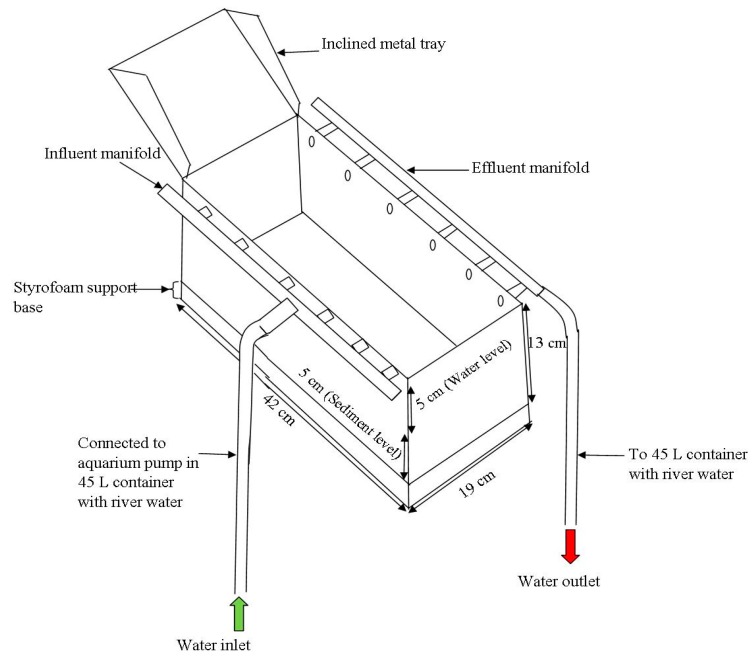
Schematic representation of sediment chambers used for the laboratory resuspension experiments (Figure modified from Abia et al. [38]).

The experiments were performed in flow-chambers (length 42 cm, width 19 cm and height 13 cm) constructed in the laboratory using periplex glass (Figure 2).

The design of the chambers was adapted from Shelton et al. [41]. The chambers were connected to aquarium pumps (maximum capacity of 600 L/h) that were submerged in 45-L plastic containers. Sediment cores (length 41.5 cm, width 18.5 cm and height 12.5 cm) were collected from three different locations in the Apies River and placed into the chambers and transported to the laboratory. These sites were selected due to the difference in their particle size distribution as previously described [42]. Site 1 (AP5) had a high clay content, Site 2 (AP6) had a high silt content while Site 3 (AP7) was mostly coarse sand. Once in the laboratory, river water collected from the same sites was added into the sediment chambers to an additional height of 5 cm. The 45-L plastic containers were also filled with river water from each site. The chambers were then inoculated by adding *E. coli* (ATCC 25922) to the overlaying water in the sediment chamber to a final concentration of ca. 107 CFU/mL. The water (and approximately the top 2-cm layer of sediments) in the sediment chamber was manually stirred using a plastic hockey stick (Thermo Fisher Scientific, Edenvale, South Africa) to allow equal distribution of the organisms in the water column and the top sediment layer. The chambers were then allowed to stand overnight at room temperature to permit settlement of the bacteria. On the sampling day, water was collected from each chamber prior to disturbing the sediments.

### 2.3. Sediment Disturbance through Mechanical Agitation

In the laboratory flow-chambers, the top 2 cm of sediments was manually stirred using a sterile disposable plastic hockey stick, ensuring that the entire length and width of the chamber was stirred to allow for proper resuspension. A new hockey stick was used for each chamber and care was taken to avoid contamination from hands during the stirring. This was to ensure that the microbial count observed in the water column was from the experiment and not from external contamination. Following the sediment disturbance, water samples were collected once the plume reached the surface. This was time zero (0 min) sampling. Thereafter, samples were collected after 10, 30 and 60 min. The water samples were analysed for *E. coli* counts using the Colilert^®^ 18/Quanti-tray^®^ 2000 system (IDEXX Laboratories (Pty) Ltd., Johannesburg, South Africa) following the manufacturer’s instructions [43]. Experiments were carried out in triplicate.

### 2.4. Sediment Disturbance through Increased Flow

For the flow experiments, a metal tray was inclined at an approximate angle of 45° perpendicular to the flow direction in the chambers (Figure 2). A sudden surge was produced by pouring approximately 3 L of river water within 30 s on the tray, thus causing a sediment disturbance in the chambers as the water from the tray reached the bottom of the chamber. The aim of this experiment was to mimic the effect of the direct discharge of large volumes of treated water from wastewater treatment works into the river. The water column was then sampled and analysed in the same way as with the mechanical disturbance experiments. The increased flow experiments were duplicated.

For both types of disturbance experiments, water turbidity was measured before and after sediment disturbance using a T100 portable turbidity meter (EUTECH Instruments, Aachen, Germany).

### 2.5. Sediment Disturbance Experiments in the Field

The manual resuspension experiment was then conducted at two selected sites on the Apies River (AP6 and AP9; Figure 1). Site A6 was selected due to the ease of accessibility and the fact that it was used by some inhabitants of the area for irrigation and personal hygiene. This was to simulate an area where community members could enter the water for recreational activities, to bathe, do laundry or for religious rituals. Site AP9 was selected because it was a point at which cows always crossed the river. Site AP9 had similar sediment particle size distribution as Site 3 (AP7) used in the laboratory experiments. At site AP6, the sediment was manually disturbed by raking an approximate 1 m^2^ area of the riverbed using a garden rake as previously described [44]. Water samples were then collected about 2 m away, downstream from the raked area to observe whether the disturbed sediments were actually carried downstream by the water current. The experiment at site AP9 was carried out at a time when cows usually cross the river. Observations made during several field visits to this sampling site showed that the farmers always watered their cows between 8:30 and 9:30 a.m. daily. It was also observed that the river water became more turbid downstream from the crossing point. Thus, the sampling point was approximately 3 m away from the point at which the cows crossed. Due to the fast-flowing nature of the water in the field experiments compared to the laboratory experiments, samples were collected at 20-s intervals from the time the plume reached the sampling point (2 m and 3 m away from the point of sediment disturbance for AP6 and AP9, respectively). Initial samples were also collected prior to mechanical disturbance. Samples were transported to the laboratory at 4 °C on ice and analysed immediately using the Colilert reagent to enumerate *E. coli*. The turbidity of the water was measured on site before and after sediment disturbance using the same instrument as for the laboratory experiments. The field experiments were performed on a single day. No direct defaecation into the water by the cows was observed during the field experiment as Site AP9.

### 2.6. Flow Conditions Necessary for Entrainment and Suspension of Sand in the Apies River

Mechanical disturbance of the riverbed is not always necessary for the mobilization of sediment and associated bacteria. Natural flows above some threshold are sufficient to initiate movement and suspend the sediment. Conventional methods have been used to determine approximate threshold conditions for the six selected sites along the Apies River where limited information has been obtained. Sediment samples were sent to the South African Agricultural Research Council for particle size analysis. Results of the sand particle sizes at these sites have previously been published [42]. The channel widths, flow depths and near-bed flow velocities were measured on one day during the dry season using a digital water velocity flow meter, the Global Water Flow Probe, Model FP211 (Global Water Instrumentation, Xylem Inc., Dallas, TX, USA).

#### 2.6.1. Entrainment

Entrainment (sometimes referred to as incipient motion), implies the mobilisation or setting in motion of riverbed material [45]. The flow condition at which entrainment would occur at each of the sites was estimated using the Shields criterion. The critical shear stress was determined for the median grain size (*d*_50_) in the bed material using the Shields diagram as compiled by Vanoni [46]. The corresponding flow depth (*D*) was then calculated from the formulation for the bed shear stress (*τ*),

(1) τ = ρ g R S

in which *ρ* is the density of water, *g* is gravitational acceleration, *R* is the hydraulic radius and *S* is the energy gradient. For wide channels, *R* can be approximated by *D* and, assuming uniform flow, *S* is equal to the channel gradient (it is acknowledged that higher energy gradients would occur during natural unsteady, non-uniform flows). The average flow velocity (*V*) corresponding to this flow depth was calculated using the Manning equation,

(2) V = 1n R23 S12

in which *n* is an empirical coefficient, estimated as 0.020 for these conditions. The discharge, or flow rate, is the product of the average velocity and the cross-sectional flow area, defined by the flow depth and channel width.

#### 2.6.2. Suspension

Once mobilised, the sediment can be transported along the bed or in suspension within the body of the flow. Suspension requires greater flow conditions than those required for entrainment, and were estimated using the criterion proposed by Nino et al. [18]. This criterion is an advance on earlier ones, such as those proposed by Bagnold [47] and van Rijn [48], being developed from van Rijn’s criterion but taking into consideration the hiding effect that sometimes occurs when smaller-sized particles are about to undergo entrainment from a rough bed [18]. The ratio of the shear velocity (u_*_) to the particle settling velocity (*w*) at the onset of suspension is determined as:

(3) $$\frac{u_{*}}{w}\;=\;21.2\;D_{*}^{-1.8}\;\;\;\mathrm{for}1<D_{*}<9$$

(4) $$\frac{u_{*}}{w}\;=\;0.4\;\;\;\mathrm{for}D_{*}>9$$

with,

(5) $$u_{*}\;=\;\sqrt{g\;D\;S}$$

(6) D∗ = ds ((Ss − 1) gν2) 13

where *d_s_* is the representative grain diameter, *S_s_* is the specific gravity of the sediment and *ν* is the kinematic viscosity of water. D_*_ = 9 corresponds to a sand size of 0.38 mm, which is smaller than all the maximum sand sizes measured at the river sites, so u_*_/w = 0.4 is the appropriate suspension condition. The settling velocity was estimated from the graph presented by Graf [49] for natural quartz grains. The flow depth corresponding to the suspension shear velocity was calculated from Equation (5) and the corresponding velocity and discharge then calculated as for the entrainment condition. The critical shear stress for suspension was calculated from the critical shear velocity using the formula:

(7) $$\tau \;=\;\rho \;u_{*}^{2}$$

### 2.7. Statistical Analysis

Data analysis was performed using SPSS 20.0 (IBM Corporation, Armonk, NY, USA). Correlation between *E. coli* count and turbidity was investigated for each sediment type using the non-parametric Spearman’s rank correlation test. The Mann–Witney U test was used to compare the turbidity as well as the *E. coli* counts after resuspension between the various sediment types. A regression analysis was used to verify if a change in turbidity (independent variable) could explain a change in *E. coli* counts (dependent variable) in water following sediment disturbance. All tests were considered statistically significant at *p* < 0.05.

## 3. Results

### 3.1. Sediment Disturbance (Mechanical)

**Figure 3:**
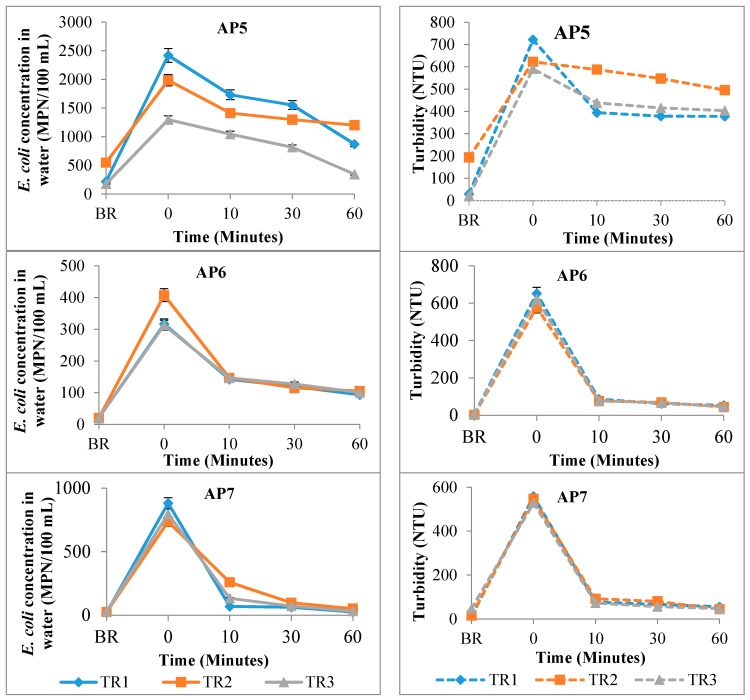
*Escherichia coli* (solid lines) and turbidity (dashed lines) results in the water column for the mechanical sediment disturbance experiment in the laboratory for the three experimental rounds (TR1–TR3); BR: before resuspension.

Mechanical disturbance of the sediment in the laboratory experiment showed a corresponding increase in water *E. coli* count and water turbidity (Figure 3).

The increase in *E. coli* in the water column following mechanical sediment disturbance ranged between 3.6 and 35.8 times higher than the initial concentration prior to sediment disturbance. The concentration of *E. coli* in the water column then gradually decreased over time to approximately the initial concentration prior to sediment disturbance. There was equally a decrease in turbidity and depending on the sediment type, the decrease was either faster or slower than the decrease in water *E. coli* counts.

### 3.2. Sediments Disturbance (Increased Flow)

**Figure 4:**
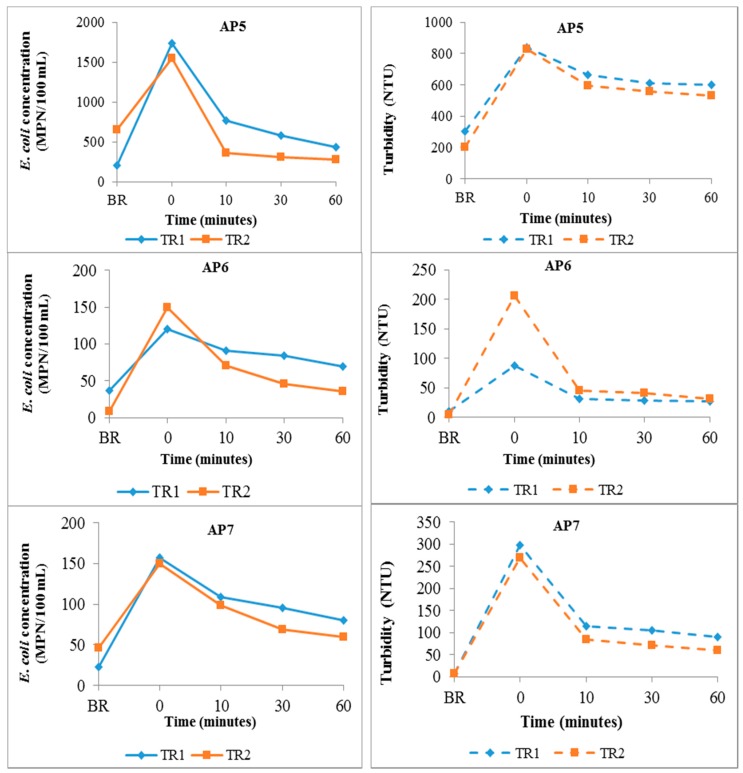
*E. coli* (solid lines) and turbidity (dashed lines) results in the water column for the sediment disturbance experiment through increased flow in the laboratory for the two experimental rounds (TR1–TR2).

The results obtained with the disturbance of bottom sediments in the chambers by increased flow are shown in Figure 4.

For the flow experiments, a 2.4- to 17.4-fold increase in *E. coli* count was observed in the water column following the induced water surge. The induced water surge also caused a peak in water turbidity as with the mechanical disturbance experiments. There was equally a strong correlation (*p* < 0.05) between the water *E. coli* counts and turbidity for all sediment types.

### 3.3. Field Sediment Disturbance Experiments

**Figure 5:**
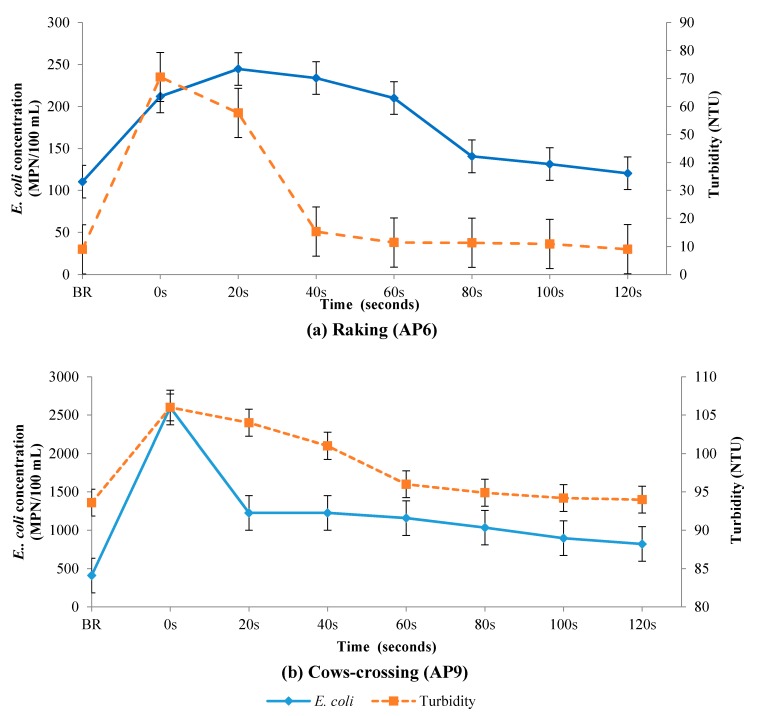
*E. coli* concentration (solid lines) and turbidity (dashed lines) of the water column before resuspension (BR), followed by (**a**) after raking and (**b**) after cow-crossing.

The field experiments consisted of raking an approximate 1 m^2^ portion of the river bed and the crossing of cows in the river. Figure 5a,b show the results of the raking and cow-crossing experiments respectively. 

Both experiments resulted in an increase in *E. coli* counts and turbidity within the water column following sediment disturbance. In the field experiment, the increase in *E. coli* was 7.9 times and 6.5 times higher than the concentration before resuspension for the raking and the cow-crossing respectively. The increase in *E. coli* concentration after sediment disturbance was positively correlated to turbidity for both experiments.

### 3.4. Correlation between E. coli and Turbidity in Sediment Disturbance Experiments

Results of the regression analysis between *E. coli* and turbidity during the laboratory and field experiments are presented in Table 1.

A positive correlation was observed between *E. coli* and turbidity for sediments of AP6 and AP7 in the laboratory experiments and at Site AP9 in the field experiment.

### 3.5. Estimation of Entrainment and Suspension for the Apies River

Due to the limited flow and river geometry data for the Apies River, the Shields criterion was used to estimate the flow conditions at which sediment particles could be entrained, and Nino et al.’s criterion [18] was used to estimate the conditions required for the sediment suspension. The measured channel widths, gradients, flow depths and near-bed flow velocities are shown in Table 2.

The sand sizes ranged from 0.18 mm (AP6) and 0.52 mm (AP8). Of all the sites studied, Site AP9 was the shallowest (0.25 m; measured at the centre of the channel width, while Site AP6 was the deepest (0.78 m). In terms of the channel width, Site AP7 was the widest (23.5 m) while Site AP2 (11.5 m) was the narrowest of all the sites. Estimated flow conditions needed for entrainment and suspension of sediments from the riverbed in the Apies River are given in Table 3.

Discharges required to just mobilise bed particles ranged between 0.004 m^3^/s (AP7) and 0.021 m^3^/s (AP8). Greater discharges ranging between 0.04 m^3^/s (AP6) and 7.23 m^3^/s (AP7) were needed to cause the particles to completely resuspend from the riverbed.

## 4. Discussion

Although several studies have been conducted on sediment resuspension within aquatic ecosystems, these have been in beach environments, mostly used for recreational activities in developed countries like the USA [50,51,52] and Portugal [53,54]. Studies reporting on sediment resuspension, especially in developing Sub-Saharan countries, are limited or not available. As per the latest United Nations’ Sustainable Development Goals, close to 700 million people (the majority of whom are in Sub-Saharan Africa) still lack access to safe portable drinking water. In these parts of the world, people still have to partially or entirely depend on surface water bodies for their daily water requirements (for drinking and other household uses). With the increased droughts experienced in many of these countries due to changing climates, the need to use these surface water bodies is expected to increase. The increased access to these water bodies could lead to increased waterborne disease outbreaks due to resuspension of microbial pathogens from sediments of such polluted water bodies. Therefore, the current study is of significance to water regulating bodies and scientists in such countries as it provides information on the need to include sediment monitoring when designing aquatic ecosystem management programmes. This would in turn help improve the quality of these aquatic environments and thus protect aquatic life in general and the health of human populations using these waters in particular.

### 4.1. Laboratory Sediments Disturbance Experiments

Once in the aquatic environment, microorganisms are exposed to several factors that can affect their ability to survive in that environment. Of these, sedimentation has been recognised as an important factor [55,56]. Although several processes have been identified as contributing to the settling of bacteria into bed sediments, attachment to suspended sediments enhances the transport of these microorganisms from the water column into the bed sediments [57,58,59]. In the sediments, the microorganisms are protected from multiple stressors like UV light and predation; and are provided with higher nutrient concentrations [24,60,61,62]. In a recent study conducted in New Zealand, Devane et al. [63] concluded that disturbing bottom sediments in an urban river resulted in the resuspension of sediment-borne microorganisms. Similar findings were obtained in our study. The disturbance of sediments in the laboratory experiments resulted in an increase in the concentration of *E. coli* in the water column (Figure 3 and Figure 4).

During the wet season, heavy rainfall may lead to erosion of riverbed sediments due to increased river flow [64]. In the laboratory flow experiments in our study, creating a sudden water surge in the flow-chambers induced a rise in *E. coli* concentration and turbidity in the water column. This observation ties in with findings of previous sediment resuspension works [23,44,65]. The correlation between the water turbidity and *E. coli* concentration suggests that in the absence of external sources, sediments could serve as a reservoir of high concentrations of microorganisms that could be resuspended, thus affecting the microbial quality of surface water bodies. The correlation between *E. coli* and turbidity also suggests that turbidity might be used as a rough, but potentially useful (local) indicator of the likelihood of microbial contamination. As such, visually observing the water for turbidity could be an immediate indication that people should avoid contact with the water. While communities may not have access to tools for testing the water for microbial quality, a visual assessment would therefore make turbidity an acceptable approach to continuously monitor the water, thus preventing possible exposure to microbial pollutants that may be present in the water.

Mechanisms that lead to weak primary bacterial attachment include attachment through van der Waals forces, electrostatic and hydrophobic interactions, hydrodynamic forces and steric hindrance [66]. When these forces are electrostatic, the negative charge of the bacterial surface and that on the non-living surface lead to a repulsive force that makes the attachment easily reversible. On the other hand, hydrophobic forces appear to be stronger [67]. Thus, during riverbed disturbances, bacteria loosely attached through the weak forces, together with bacteria in the inter-grain spaces are released into the water column and can be detected. Also, it has been reported that bacteria will attach faster to sediments with smaller grain sizes [68]. This could explain the higher increase in *E. coli* concentration in the water column of chambers containing sediments from Site 1 (AP5) with higher clay content (14.5%) compared to Site 2 (AP6; 7.8%) and Site 3 (AP7; 2.6%) which had higher medium to coarse sand particles. The colloidal nature of the clay particles also allowed *E. coli* to stay in the water column for a longer period of time. The larger sizes and thus heavier particles of sediments from site AP6 and AP7 favoured a rapid settling of the particles together with the attached bacteria. This, however, indicates that in some places that are dominated by clay materials, smaller particles may settle slower than bound bacteria, thus resulting in low correlation between microbial counts and turbidity.

### 4.2. Field Sediment Disturbance Experiments

The Apies River has several uses. Towards the northern end of the river before it joins the Pieenaars River, the Apies River is the main source of water for many farmers that use its waters for their animal farms. It is also used by households as a point for laundry usually involving the women and children getting into the water to do washing. The field study was conducted to mimic sediment disturbance during such water uses. Samples collected approximately 2 m from the sediments disturbance point revealed an increase in *E. coli* concentration in the water column. The increase in the *E. coli* concentration obtained in this study following sediments disturbance are similar to those reported by Orear and Dalman [44] who recorded a 7.5-fold increase in the concentration of *E. coli* in the water column following a 30-s raking of a 1-m^2^ plot. Recreational activities have been reported to cause resuspension of sediment-borne microorganisms [69]. It should be noted, however, that the degree of resuspension during recreational or other in-stream activities may also depend on the number of persons involved and the total area of the riverbed disturbed.

During the dry winter season, most farmers walk their cows to the river so that they can drink directly from the river water. The crossing of the cows in the rivers results in the disturbance of the sediments in the riverbed which leads to the resuspension of sediment-borne organisms as shown in the results of the field experiments reported in this study. The effect of cows crossing a river on the concentration of *E. coli* in the water column has previously been reported [70,71]. However, contrary to the studies of Davies–Colley et al. [62] and of McDaniel and Soupir [63], no direct defaecation by the cows was observed during the current study. This therefore means that the increase in *E. coli* concentration and the turbidity recorded during the cows-crossing experiment was solely due to the resuspension of the sediments from the riverbed. The Apies River is also used for rituals by some religious groups. These religious groups believe that the water and sediments from the river are a source of protection and so use the river for spiritual cleansing and spiritual fortification of their homes (personal communication with a villager who came to collect water and sediments from the river). Such activities and other recreational activities could therefore lead to the resuspension of microorganisms from sediments and could represent a possible health risk to the users.

### 4.3. Correlation between E. coli and Turbidity during the Resuspension Experiments

Some studies have reported a strong positive correlation between *E. coli* concentrations and turbidity in water bodies [72,73]. The increase in *E. coli* concentration in the water column both in the laboratory and field sediment disturbance experiments in the current study, coincided with an increase in the water turbidity, demonstrated by a strong correlation between the two parameters. These results support the findings of Muirhead et al. [74] and Walters et al. [24]. In both their studies, they observed that following sediment disturbance, *E. coli* concentrations and turbidity (or total suspended solids) in the water column showed a positive correlation. In a study conducted in the city of Las Vegas, NV, USA, Huey and Meyer [73] reported that faecal indicator bacteria (*E. coli* and *Enterococci* spp.) were strongly correlated to turbidity. The authors concluded that turbidity could be used as an indicator of the microbial quality of water bodies. Although this has been shown, George et al. [75] reported that the relationship between *E. coli* and turbidity could be affected by the size of the watershed involved. In the current study, although sediment disturbance resulted in an increase in *E. coli* concentrations and turbidity values, this did not always result in a positive correlation between the two parameters (Table 1). While a strong *E. coli*/turbidity correlation was observed in some sediment types (AP6 and AP7) in the laboratory experiments and AP9 in the field experiments, *E. coli*/turbidity correlation was not statistically significant at Site AP5 in the laboratory and AP6 in the field. This suggests that sediment characteristics are important factors with regard to the correlation between *E. coli* and turbidity. In the sediments of Site AP5 that had the highest clay composition, attached *E. coli* could have settled faster while free colloidal clay particles remained in the water column for a longer period. This could have therefore led to the turbidity remaining higher even when the *E. coli* concentration had reduced to approximately initial values. On the other hand, in the sediments of AP7 (and AP9 in the field) that was made up of over 90% sand particles, the bound bacteria settled together with the heavier sand particles, resulting in the strong positive correlation observed between turbidity and *E. coli*. In a real river setting where flow is not controlled as in the laboratory experiments, other factors could interfere with the turbidity beyond the point of sediment disturbance, thus affecting the correlation [21]. Thus, Tornevi et al. [76] observed that the ease of measuring turbidity makes it a suitable first line parameter for estimating levels of microbial contamination. The authors, however, pointed out that because turbidity could be affected by organic and inorganic particles loads, it was necessary to also measure faecal indicator densities to complement the turbidity results.

### 4.4. Estimation of Entrainment and Suspension for the Apies River

The flow regime of a river as well as its geometry and sediment properties affect the deposition and resuspension of sediments from the riverbed. However, sediment resuspension (and attached microorganisms) from a riverbed is a complex process and has been shown to occur even during normal or base flow conditions [69,70]. In the present study, all the sites for which estimation (entrainment and suspension conditions) was done were characterised by a high (72%) to a very high (86%) proportion of sandy material of various sizes [42] and thus it was assumed that the largest particle sizes (represented by the largest sieve size of 0.5 mm for site AP6 and 2 mm for all the other sites) needed to be suspended. Also, river measurements (width, depth and near-bed velocity) were only taken on one day during the dry season, i.e., on 6 June 2014. The threshold flow velocities, as calculated above, are depth-averaged values (approximately equal to the local velocity at 60% of the total depth below the water surface). The water velocity varies in an approximately logarithmic manner along a vertical profile, from almost zero at the riverbed to a maximum close to the surface [77]. The measured velocities in the current study were close to the bed, and therefore cannot be compared directly with the average threshold velocities calculated for entrainment and suspension. A more reliable comparison is between the actual bed shear stress (calculated from the measured flow depth and slope through Equation (1)) with the threshold values. These have been included in Table 2 and Table 3 and show that entrainment would be expected at all sites on the measurement day, with suspension at sites AP1, AP2 and AP6 and near suspension at sites AP7, AP8 and AP9. Considering that the measurements were taken in the dry season, mobilisation and resuspension of sediment and associated bacteria are likely to occur during normal flow conditions in the Apies River.

## 5. Conclusions

Using simple laboratory and field-based experiments, the present study investigated the impact of riverbed sediment disturbances on the resuspension of *Escherichia coli* from riverbed sediment into the water column of the Apies River. The study also estimated flow conditions under which sediment particles within the Apies River could be resuspended. We conclude that sediment disturbance leads to resuspension of riverbed sediments and associated microorganisms and that this resuspension is likely to have a negative effect on the microbial quality of the overlying water. The strong correlation between *E. coli* and turbidity observed in this study suggests that turbidity could be a suitable proxy which communities may use as a first line of evidence of the possible poor quality of the river and therefore not use the water during such times. Under appropriate flow conditions, sediments in the Apies River would be resuspended and could represent a potential health risk to populations using the river directly without treatment for recreation and other purposes. Although the criteria used and hence the flow conditions obtained for entrainment and suspension in this study are empirical and assumptions have been made in their estimation, the results provide simple evidence of the effect of sediment resuspension on the microbial quality of the water in the Apies River. It is recommended that a complete profiling (e.g., the actual discharge, cross-sectional velocity, the river gradient) of the Apies River be undertaken. Obtaining measured values of these parameters may help in a better understanding of the sediment (and hence sediment-borne bacteria) dynamics within the catchment leading to more appropriate water quality monitoring strategies for the river.

## Figures and Tables

**Table 1:** Correlation coefficients and significance values for the regression analysis between *E. coli* and turbidity in the laboratory and field experiments.

Experiment	AP5 (High Clay)			AP6 (High Silt)			AP7 (High Sand)		
Lab Manual	R1	R2	R3	R1	R2	R3	R1	R2	R3
*R*^2^	0.867	0.840	0.694	0.894	0.885	0.888	0.995	0.956	0.994
*p*-value	0.022 *	0.029 *	0.080	0.015 *	0.017 *	0.017 *	0.000 *	0.004 *	0.000 *
Lab Flow	R1	R2		R1	R2		R1	R2	
*R*^2^	0.748	0.256		0.793	0.942		0.893	0.922	
*p*-value	0.058	0.385		0.043 *	0.006 *		0.015 *	0.009 *	
Field	AP6	AP9							
*R*^2^	0.383	0.673							
*p*-value	0.102	0.013 *							

TR1–TR3: experimental rounds (replicates); *: *p* < 0.05; AP5, AP6, AP7: Sediment and water collection sites for laboratory experiments; AP6 (raking site) and AP9 (cow-crossing site): Field experiment.

**Table 2:** River parameters measured at the sampling sites on the Apies River.

Site	Median Sand Size (mm)	Channel Gradient	Width (m)	Depth (m)	Bed Shear Stress (N/m^2^)	Near-Bed Flow Velocity (m/s) *
AP1	0.46	0.0040	12.0	0.45	18	0.60
AP2	0.43	0.0040	11.5	0.45	18	0.20
AP6	0.18	0.0030	14.4	0.78	23	0.30
AP7	0.5	0.0020	23.5	0.28	5.5	0.40
AP8	0.52	0.0020	11.7	0.27	5.3	0.60
AP9	0.25	0.0020	14.5	0.25	4.9	0.30

* Measured at the centre of the river cross-section. AP1, 2, 8: Additional river sites which were accessed by the inhabitants for bathing and other purposes.

**Table 3:** Estimated sediment mobilisation and suspension flow conditions for sampling sites on the Apies River.

Site	Mobilisation				Suspension			
	Depth (m)	Velocity (m/s)	Bed Shear Stress (N/m^2^)	Discharge (m^3^/s)	Depth (m)	Velocity (m/s)	Bed Shear Stress (N/m^2^)	Discharge (m^3^/s)
AP1	0.006	0.11	0.25	0.008	0.15	0.90	5.96	1.64
AP2	0.006	0.10	0.24	0.007	0.15	0.90	5.96	1.57
AP6	0.006	0.09	0.17	0.008	0.02	0.18	0.50	0.04
AP7	0.013	0.12	0.26	0.004	0.30	1.01	5.96	7.23
AP8	0.014	0.13	0.27	0.021	0.30	1.01	5.96	3.60
AP9	0.009	0.10	0.18	0.013	0.30	1.01	5.96	4.46
